# Pigs that recover from porcine reproduction and respiratory syndrome virus infection develop cytotoxic CD4^+^CD8^+^ and CD4^+^CD8^-^ T-cells that kill virus infected cells

**DOI:** 10.1371/journal.pone.0203482

**Published:** 2018-09-06

**Authors:** Chungwon J. Chung, Sang-Ho Cha, Amanda L. Grimm, Dharani Ajithdoss, Joanna Rzepka, Grace Chung, Jieun Yu, William C. Davis, Chak-Sum Ho

**Affiliations:** 1 Department of Veterinary Microbiology and Pathology, Washington State University, Pullman, Washington, United States of America; 2 VMRD Inc., Pullman, Washington, United States of America; 3 Animal and Plant Quarantine Agency, Gimcheon, Republic of Korea; 4 Gift of life Michigan, Ann Arbor, Michigan, United States of America; Universitat de Lleida, SPAIN

## Abstract

Porcine reproductive and respiratory syndrome virus (PRRSV) infection is difficult to control because the virus undergoes antigenic variation during infection and also modulates the protective host immune response. Although current vaccines do not provide full protection, they have provided insight into the mechanisms of protection. Live PRRSV vaccines induce partial protection before the appearance of neutralizing antibody, suggesting cell-mediated immunity or other mechanisms may be involved. Herein, we demonstrate recovery from infection is associated with development of cytotoxic T-lymphocytes (CTL) that can kill PRRSV-infected target cells. Initial experiments showed survival of PRRSV-infected monocyte derived macrophage (MDM) targets is reduced when overlaid with peripheral blood mononuclear cells (PBMC) from gilts that had recovered from PRRSV infection. Further studies with PBMC depleted of either CD4^+^ or CD8^+^ T-cells and positively selected subpopulations of CD4^+^ and CD8^+^ T-cells showed that both CD4^+^ and CD8^+^ T-cells were involved in killing. Examination of killing at different time points revealed killing was biphasic and mediated by CTL of different phenotypes. CD4^+^CD8^+high^ were associated with killing target cells infected for 3–6 hours. CD4^+^CD8^-^ CTL were associated with killing at 16–24 hours. Thus, all the anti-PRRSV CTL activity in pigs was attributed to two phenotypes of CD4^+^ cells which is different from the anti-viral CD4^-^CD8^+^ CTL phenotype found in most other animals. These findings will be useful for evaluating CTL responses induced by current and future vaccines, guiding to a novel direction for future vaccine development.

## Introduction

Porcine reproductive and respiratory syndrome (PRRS) is one of the most important porcine diseases with a major economic impact, causing more than $600 million per year of direct loss in the USA [1,2]. PRRS virus is in the genus arterivirus and family *arteriviridae*, and has a single strand ~15 Kb RNA genome encoding at least 10 open reading frames (ORFs 1a, 1b, 2a, 2b, 3, 4, 5, 5a, 6 and 7) [3,4]. Increasing genetic diversity among recently isolated PRRSVs [3,5,6] and the emergence of highly pathogenic PRRSVs in different geographical regions [7–18] present a tremendous challenge to the control of PRRSV using currently available vaccines. Two conventional vaccine formats, inactivated and live attenuated virus are currently in use, but neither of the vaccines induce satisfactory protection [19–21]. Inactivated PRRSV vaccines induce weak neutralizing antibody responses against only homologous strains of PRRSV, and thus have poor efficacy in porcine herds infected with genetically diverse variants of PRRSV [22]. Live attenuated PRRSV vaccines are reported to contribute to the reduction of PRRS diseases without preventing infection. Although potentially useful, the high probability of mutation resulting in reversion to virulence in vaccinated herds has been debating as a concern on use of attenuated viruses as a vaccine [8,23,24]. Therefore, there is a need to develop safer and more efficacious vaccines that induce protective immune responses against diverse PRRSVs.

Correlates of adaptive immune protection against PRRSV need to be systematically defined in PRRSV-infected and vaccinated pigs. Passive transfer of hyper-immune anti-PRRSV serum with neutralizing antibody activity to naïve pigs contributed to reducing the viral load in plasma in comparison with controls challenged with the homologous strain of PRRSV. However, presence of passively transferred antibody did not affect viral replication in tissues [25]. Low titer neutralizing antibodies produced in infected pigs were not able to control infection with PRRSV [26–29]. This weak potency of neutralizing antibodies was also restricted to homologous virus in PRRSV-infected pigs, limiting the potential for inducing protection against heterologous virus challenge [30]. Several studies have reported that immunization with live attenuated and killed virus vaccines reduced clinical disease and/or viremia after PRRSV infection before the appearance of neutralizing antibody in serum, suggesting appearance of other mechanisms of protection early in the course of infection [31–34]. Cell-mediated immunity (CMI) has been proposed as a protective mechanism in vaccinated pigs [34–37]. Studies to date have identified T-cell epitopes in several PRRSV proteins (e.g., M, N, GP3, GP4, GP5, NSP2, NSP5 and NSP9) using cytokine-based assays and synthetic peptides to stimulate effector cells [36,38–43]. T-lymphocytes that recognize these epitopes could be providing protection through targeted killing of infected cells in tissues that have not been cleared by neutralizing antibodies. However, there has been no demonstration that specific porcine T-lymphocytes can kill PRRSV-infected cells. Therefore, approaches are needed to define the relative contribution of mechanisms of adaptive immunity, such as CMI, which could form the basis for future vaccine development, aimed at providing immune protection against diverse PRRSVs.

Multiple CMI assays, such as interferon gamma enzyme-linked immunosorbent spot (ELISpot) assay and intracellular staining (ICS) assay of cytokines, have been used to evaluate the immune response to PRRSV [33,34,38]. Assays detecting cytokines as the readout can evaluate an early activation step of PRRSV-specific T-lymphocytes, but cannot reveal development of terminal antiviral activity, such as the cytolysis of PRRSV-infected cells or suppression of PRRSV replication in target cells. The inability to directly measure protective mechanisms against virus infection is a major drawback to using cytokine assays to evaluate immune responses [44,45]. The proliferation of T-lymphocytes as the readout of PRRSV-specific CMI responses may be a better approach for detection of effector function than cytokine production. However, the limitations of proliferation-based T-lymphocyte assays have been lower sensitivity/specificity and the need for use of radioisotopes to increase the sensitivity. Thus, assays for measuring terminal CMI effector functions in PRRSV-infected target cells will be critical for defining the presence and efficacy of PRRSV-specific CMI responses.

Two assays were developed in the present study, a virus suppression assay which measures the reduction of PRRSV-infected target cells mediated by effector cells and a cytotoxic T-lymphocyte (CTL) assay that measures the direct killing of PRRSV-infected cells, using monocyte-derived macrophages (MDMs) as target cells and T-cells as effector cells. These assays were used to test the hypothesis that recovery from clinical PRRS disease is associated with development of CTL with the capacity to kill PRRSV-infected cells.

## Results

### Peripheral blood mononuclear cells (PBMC) from gilts that had recovered from infection by PRRSV reduce the number of PRRSV-infected MDM

**Fig 1:**
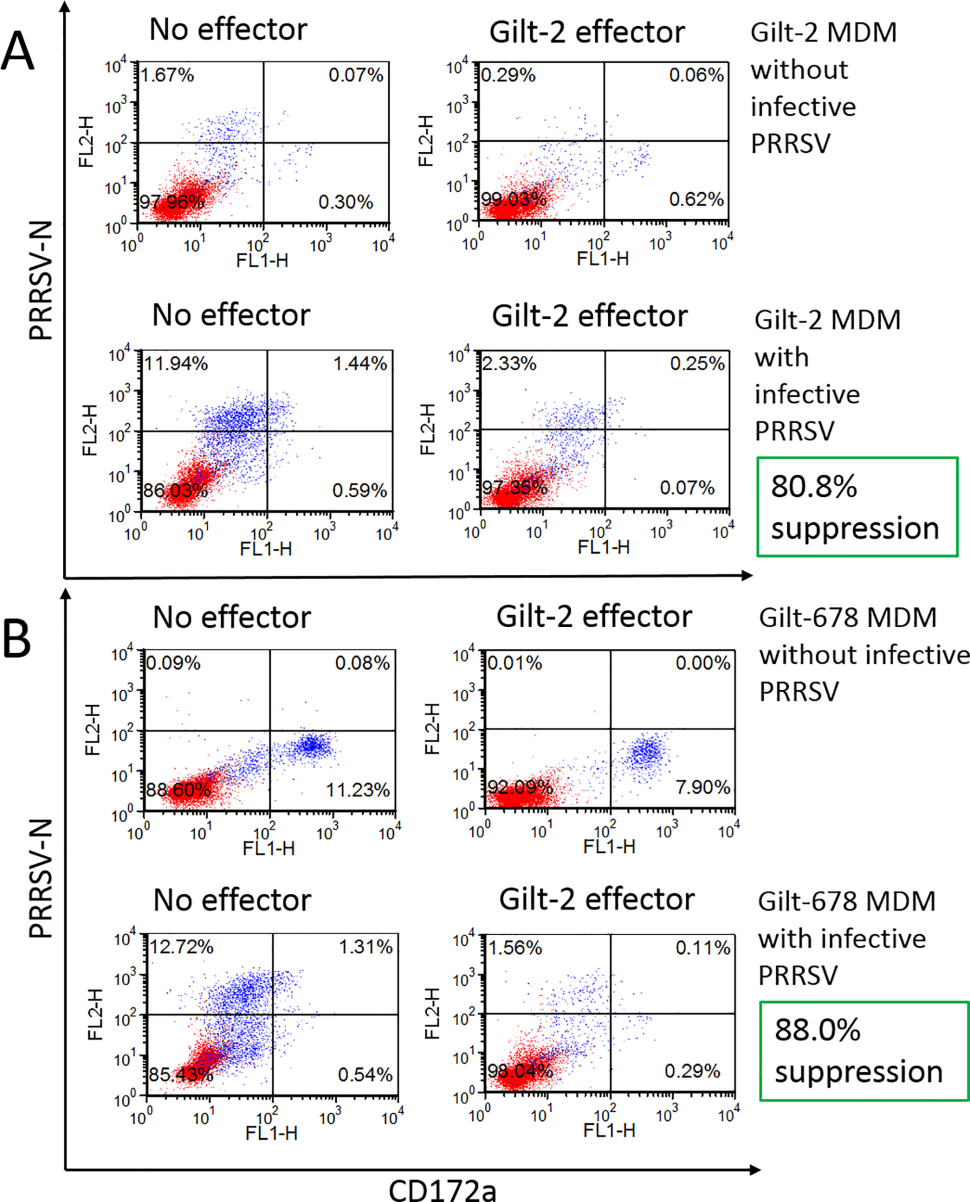
**Reduction of PRRSV**_**SD23983**_ **replication in autologous (gilt-2) MDM (A) and MHC-matched heterologous (gilt-678) MDM (B) by PBMC from PRRSV-challenged and clinically recovered gilt-2.** Effector PBMC stimulated with heat-inactivated PRRSV for 7 days were added to the virus suppression assay wells containing MDM with (MOI 0.00005) or without infective PRRSV and incubated for 6 days. X axis (CD172a) indicates MDM target cells with CD172a marker stained with CD172a-specific monoclonal antibody and Y axis (PRRSV-N) indicates PRRSV-infected target cells stained with PRRSV N protein-specific monoclonal antibody. Suppression of PRRSV replication was calculated as the percent reduction of all PRRSV-infected MDM in the presence of effector cells as compared to PRRSV-infected MDM alone.

**Fig 2:**
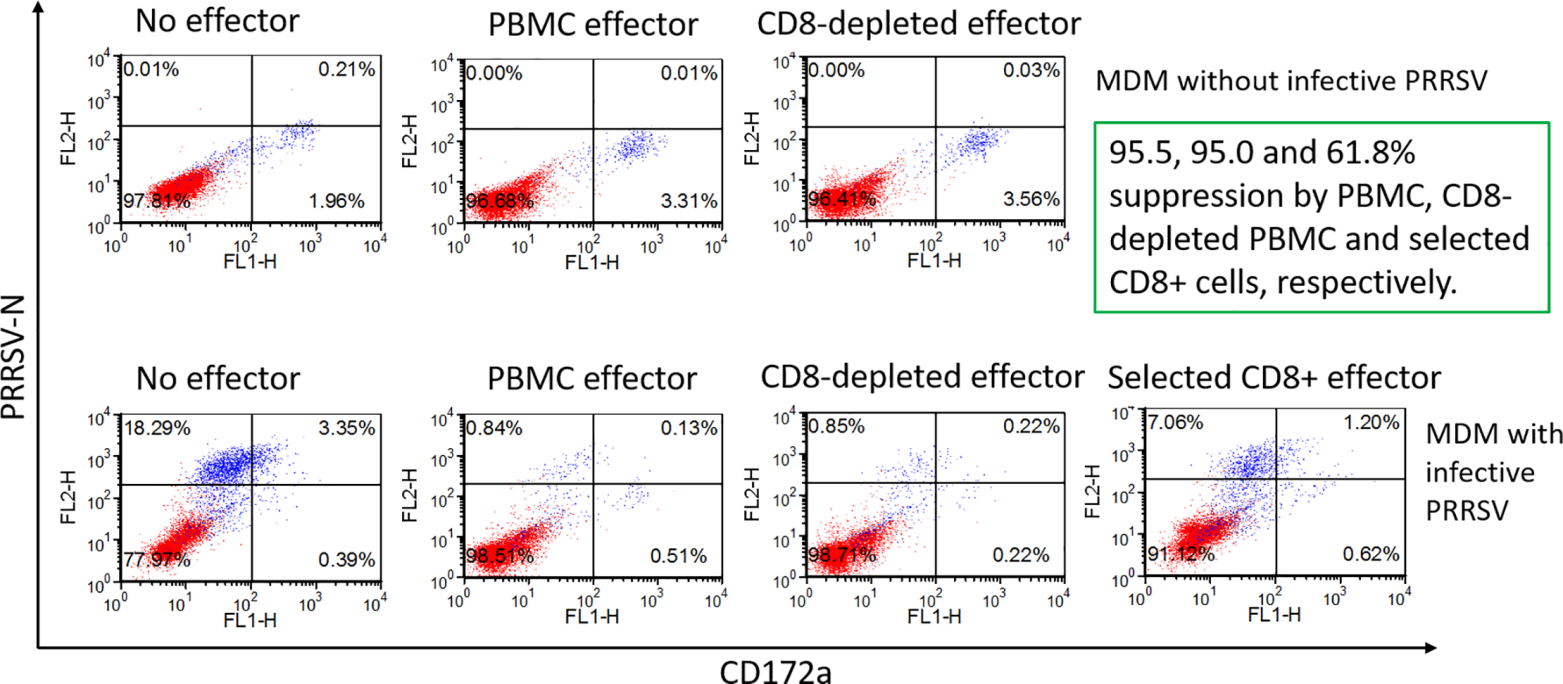
Reduction of PRRSV_SD23983_ replication in autologous (gilt-2) MDM by non-CD8^+^ T-cells in PBMC from gilt-2. Effector cells stimulated with heat-inactivated PRRSV for 7 days were added to the virus suppression assay wells containing MDM with (MOI 0.00005) or without infective PRRSV to incubate for 6 days. The suppression of PRRSV replication was calculated by percent reduction of the PRRSV-infected MDM in the presence of effector cells as compared to target cells alone.

A flow cytometry-based virus suppression assay was developed initially to determine whether the reduction in virus replication in MDM target cells is mediated by specific effector cells that disrupt replication by killing the infected cells. Gilt-2 PBMC effector cells at 5:1 effector to target ratio reduced autologous PRRSV-infected MDM in one experiment by 80.8% (Fig 1A) and in another experiment by 95.5% (Fig 2, lower panel, gilt-2 PBMC effector). Gilt-2 PBMC also caused an 88.0% reduction of PRRSV-infected MDM from a MHC-matched heterologous uninfected gilt-678 (Fig 1B).

**Fig 3:**
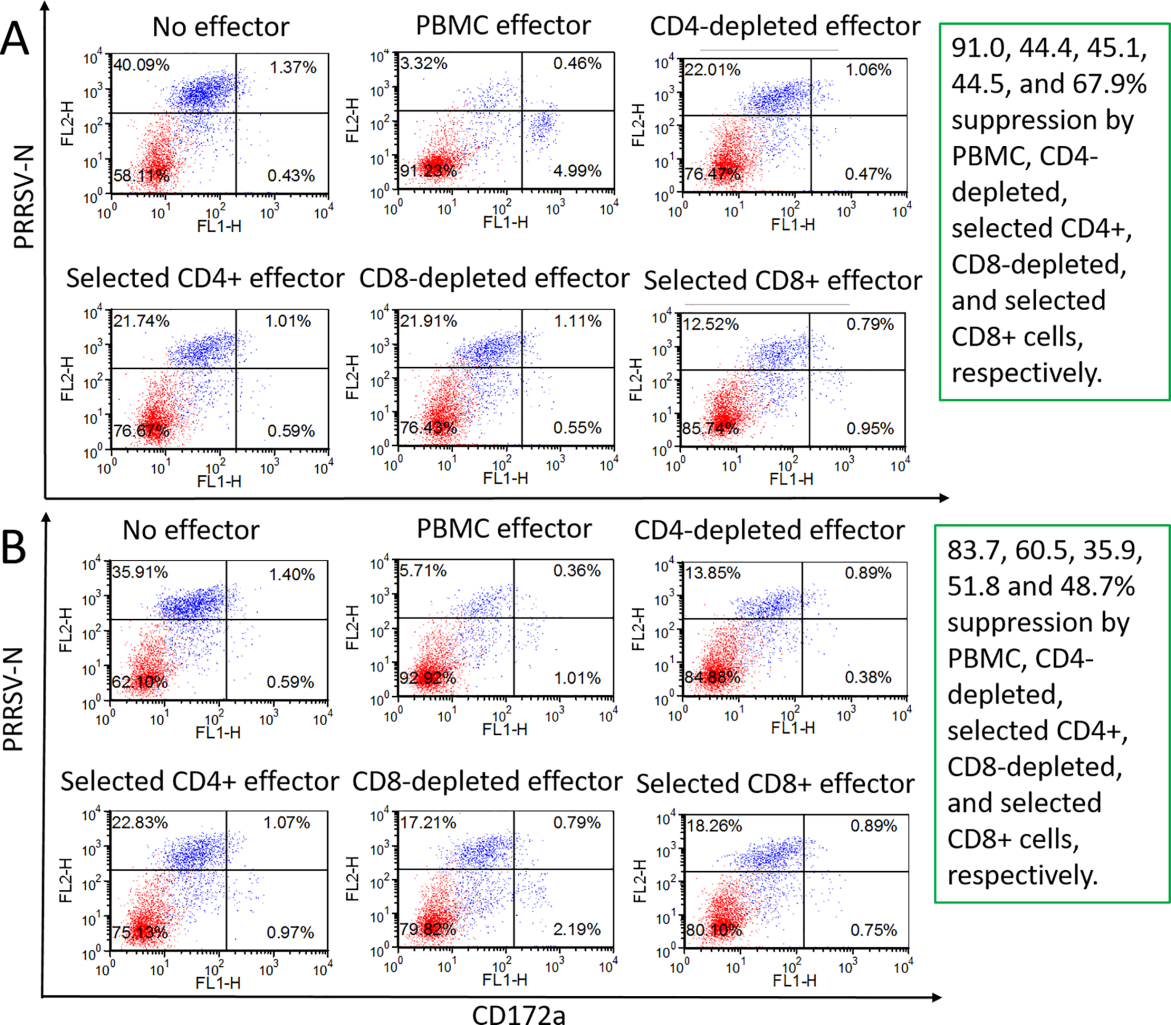
**Reduction of PRRSV**_**SD23983**_ **replication in MHC-matched heterologous (gilt-676) MDM by PBMC and their subpopulations from two PRRSV-challenged and clinically recovered gilts (A. gilt-1 and B. gilt-3).** Effector cells stimulated with heat-inactivated PRRSV for 7 days were prepared as total PBMC, CD8^+^ T-cell-depleted or selected PBMC, CD4^+^ T-cell-depleted or selected PBMC, added to the virus suppression assay wells containing MDM with infective PRRSV (MOI 0.00005), and then incubated for 6 days. The suppression of PRRSV replication was calculated by percent reduction of the PRRSV-infected MDM in the presence of effector as compared to target cells alone.

The suppression assay was also used to evaluate PBMC from two other PRRSV-infected gilts (gilt-1 and gilt-3) with another MHC haplotype (Class I = Lr-32.0/35.0, Class II = Lr-0.1/0.12) and MDM target cells from non-infected MHC-matched gilt-676. Gilt-1 and gilt-3 PBMC stimulated with MDM, pulsed with heat-inactivated PRRSV, reduced PRRSV-infected MDM by 91.0% and 83.7%, respectively (Fig 3A top panel, gilt-1 PBMC effector; Fig 3B top panel, gilt-3 PBMC effector). The effective reduction of PRRSV-infected MDM by gilt-1 and gilt-3 PBMC with a different MHC haplotype demonstrated that virus suppression was not limited to a particular MHC haplotype of pigs.

### Identification of T-cell subpopulations causing reduction of PRRSV-infected MDM

The initial experiments with the suppression assay were conducted with effector cells from gilt-2, using total PBMC, CD8^+^ T-cell-depleted PBMC, and positively selected CD8^+^ T-cells. Both total PBMC and CD8^+^ T-cell-depleted PBMC (at 5:1 effector to target ratio) reduced the number of PRRSV-infected MDM by 95% (Fig 2, lower panel; compare PBMC effector with CD8^+^ T-cell-depleted effector). This result indicated that considerable anti-PRRSV efficacy in gilt-2 was attributable to non-CD8^+^ effector cells in PBMC, possibly CD4^+^CD8^-^ T cells. However, there was still a 61.8% reduction of infected MDM using isolated CD8^+^ T-cells from gilt-2 at 0.5:1 effector to target ratio (Fig 2, lower panel; positively selected CD8^+^ effector cells).

The relative role of CD4^+^ and CD8^+^ T-cells in the suppression assay was investigated further, using PBMC, CD4^+^ and CD8^+^ subpopulations from gilt-1 and gilt-3. Total PBMC and PBMC depleted of CD4^+^ or CD8^+^ cells, at 5:1 effector to target ratio, and positively selected CD4^+^ and CD8^+^ T-cells, effector to target ratio at 0.5:1, reduced PRRSV-infected MDM by 91.0, 44.4, 44.5, 45.1 and 67.9%, respectively (Fig 3A). Results similar to gilt-1 were obtained with gilt-3, using identical cell preparations. There was an 83.7, 60.5, 51.8, 35.9, and 48.7% reduction in infected target cells, respectively (Fig 3B). Depleting CD8^+^ T-cells in PBMC from these two gilts removed more of the PRRSV suppressive activity than did the same treatment of PBMC from gilt-2 (Fig 2 lower panel, CD8^+^ T-cell-depleted effector). However, both positively selected CD4^+^ and CD8^+^ T-cells from gilt-1 and gilt-2, at a low effector to target ratio (0.5:1), significantly reduced PRRSV-infected MDM, demonstrating a contribution by both of these T-cell subpopulations in the suppression assay.

### PBMC with PRRSV suppressive activity have CD4^+^ and CD8^+^ T-cells expressing granzyme-B and kill infected MDM

**Fig 4:**
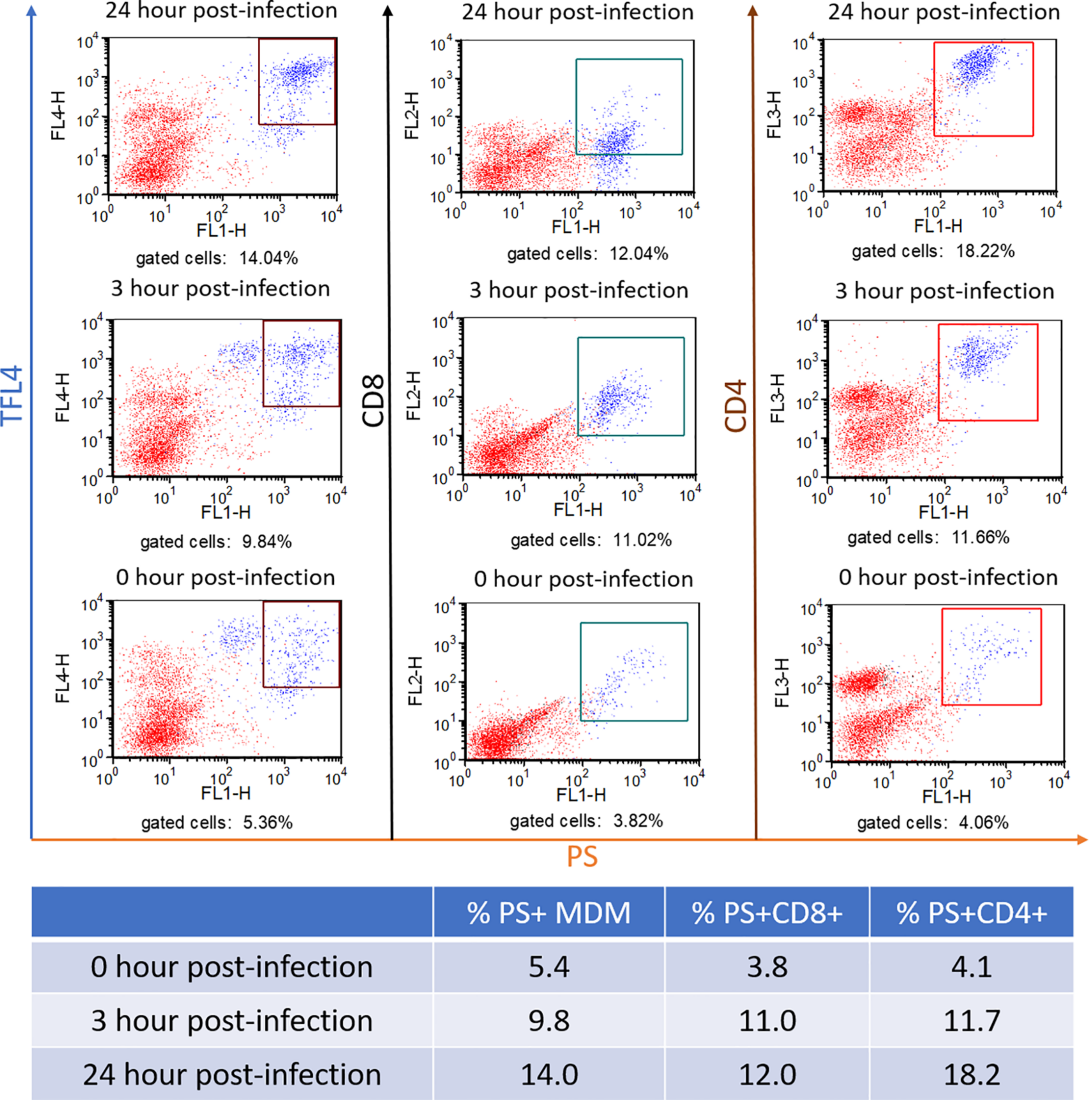
PRRSV_SD23983_-infected and clinically recovered gilt-2 had PRRSV-specific cytotoxic T-cells. Cytotoxic T-lymphocyte responses were measured by the percentage of PRRSV-infected MDM (TFL4^+^) that received the lethal death signal (PS^+^) at 0, 3 and 24 hours post-PRRSV infection. The phenotypes of cytotoxic T-cells were determined by the percentage of CD8^+^ or CD4^+^ T-cells that produced the lethal death signal (PS^+^) after 1-hour incubation with MDM infected with PRRSV for 0, 3 and 24 hours.

The data obtained from the suppression assay suggested that suppression was mediated by direct killing of infected target cells by cytotoxic T-lymphocyte (CTL). To explore this possibility, a flow cytometric assay was developed, based on the use of fluorescent dye (TFL4) that labels the infected target cells and a fluorogenic PanToxiLux substrate (PS) for granzyme B and upstream caspase [46,47]. With the use of a selective gate placed on TFL-4^+^ target cells, dead target cells could be distinguished from live target cells by detection of enzymatically activated PS in dead cells. PBMC, from gilt-2, stimulated with heat-inactivated PRRSV_SD23983_ for 7 days were reacted with MDM infected with PRRSV_SD23983_ at a multiplicity of infection (MOI) of 19.3 for 24-hours. Sets of infected target cells were analyzed at 0, 3, 6, 12, 18 and 24-hours post-infection. The CTL delivery of lethal hit (death signals detected by PS^+^) to PRRSV-infected MDM was detected by enumerating target (TFL4^+^) cells that were PS^+^ by flow cytometry. TFL4^+^PS^+^ cells had cleaved fluorogenic substrates for granzyme-B and upstream caspases which is a reliable measure of target cell killing by CTL [46,47]. Killing was demonstrated as the percentage TFL4^+^PS^+^ MDM was 14.04% at 24-hour post-infection (hpi) with PRRSV, which was much higher than the 5.38% in the MDM at 0 hpi (Fig 4, left three panels). Furthermore, gilt-2 CTL also recognized epitopes presented on PRRSV-infected MDM at 3 hpi which was possibly before *de novo* synthesis of viral proteins. This cytotoxicity caused a 1.8-fold (82%) increase in MDM containing death signals (TFL4^+^PS^+^) between 3 hpi (9.84%) and 0 hpi (5.36%) (Fig 4, left panels). That PRRSV-infected target cells were killed before *de novo* synthesis of PRRSV proteins indicated that virus epitopes were processed and presented from PRRSV incoming into MDM by the exogenous pathway.

The T-cell phenotypes activated by PRRSV-infected MDM were determined by the percentage of CD4^+^PS^+^ and CD8^+^PS^+^. PS^+^ T-cells likely cleaved the fluorogenic substrate predominantly with granzyme-B, and not upstream caspases, since only live T-cells were gated for analysis. Expression of granzyme-B in T-lymphocytes is necessary for delivery to and killing of target cells [47]. CD4^+^PS^+^ T-cells had much higher percentages after interaction with MDM at 3 hpi (11.7%) and 24 hpi (18.22%) than 0 hpi (4.06%) (Fig 4, right panels). Similarly, CD8^+^PS^+^ T-cells were increased at 3 hpi (11.0%) and 24 hpi (12.04%) compared to 0 hpi (3.82%) (Fig 4, center panels). These results demonstrated that both CD4^+^ and CD8^+^ gilt-2 T-cells expressed granzyme-B while killing PRRSV-infected MDM (Fig 4, left panels).

### Different T-cell subpopulations had unique recognition patterns of PRRSV-infected MDM

**Fig 5:**
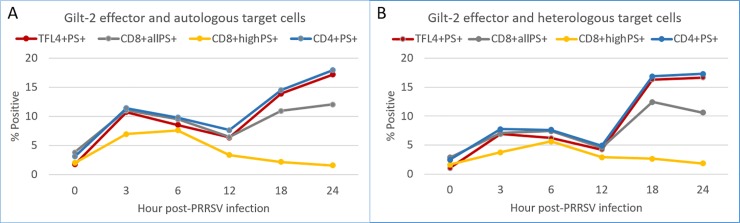
Evaluation of CD4^+^, CD8^+all^ and CD8^+high^ CTL recognizing epitopes on MDM infected with PRRSV for 0 to 24 hours. CTL activity was measured as the percentage of PRRSV-infected MDM (TFL4^+^) having death signals (PS^+^) at 0, 3, 6, 12, 18 and 24 hours post-PRRSV infection. The phenotypes of CTL effectors were determined by the percentage of CD8^+^ or CD4^+^ T-cells having death signals (PS^+^) after 1-hour incubation with MDM infected with PRRSV for 0, 3, 6, 12, 18 and 24 hours. Autologous and heterologous MHC-matched MDMs were prepared from PBMC of gilt-2 and gilt-678, respectively.

To determine the appearance of CTL epitopes during cell infection and the pattern of recognition and activation by CD8^+high^PS^+^, CD8^+all^PS^+^ and CD4^+^PS^+^ T-cells, the CTL assay was carried out using MDM infected for 0 to 24 hours. The same 7-day-stimulated gilt-2 PBMC effectors were used for each time point of the assay. The percentage of autologous PRRSV-infected MDM with death signals (TFL4^+^PS^+^) was biphasic with a moderate peak (10.7%) at 3 hpi followed by a drop at 12 hpi (6.3%), and a second, major peak starting at 18 hpi (13.9%) to 24 hpi (17.1%) (Fig 5A). Similar results were obtained with heterologous, MHC-matched, PRRSV-infected MDM (Fig 5B). Together these results demonstrated that CTL epitope expression varied in MDM over 24 hpi, as the same effector cells were used for each time point.

The highest percentages of activated CD8^+high^PS^+^ T-cells were at 3 and 6 hpi with a subsequent decrease so that by 24 hpi the percentage was the same as 0 hpi (Fig 5A & 5B). This monophasic response of CD8^+high^PS^+^ T-cells was in contrast to the biphasic recognition that occurred with the CD4^+^PS^+^ CTL (Fig 5A & 5B); a peak was observed at 3 hpi and another higher peak at 24 hpi (Fig 5A & 5B). Interestingly, the percentage of CD4^+^PS^+^ T-cells coincided with the percentage of MDM receiving the death signals (TFL4^+^PS^+^) at all tested time points (Fig 5A & 5B). Unlike the CD8^+high^PS^+^ T-cells, the CD8^+all^PS^+^ T-cells had a biphasic response more like the CD4^+^PS^+^ T-cells (Fig 5A & 5B).

### Contribution of CD8^+^ T-cells to killing PRRSV-infected MDM early in the virus replication cycle

**Fig 6:**
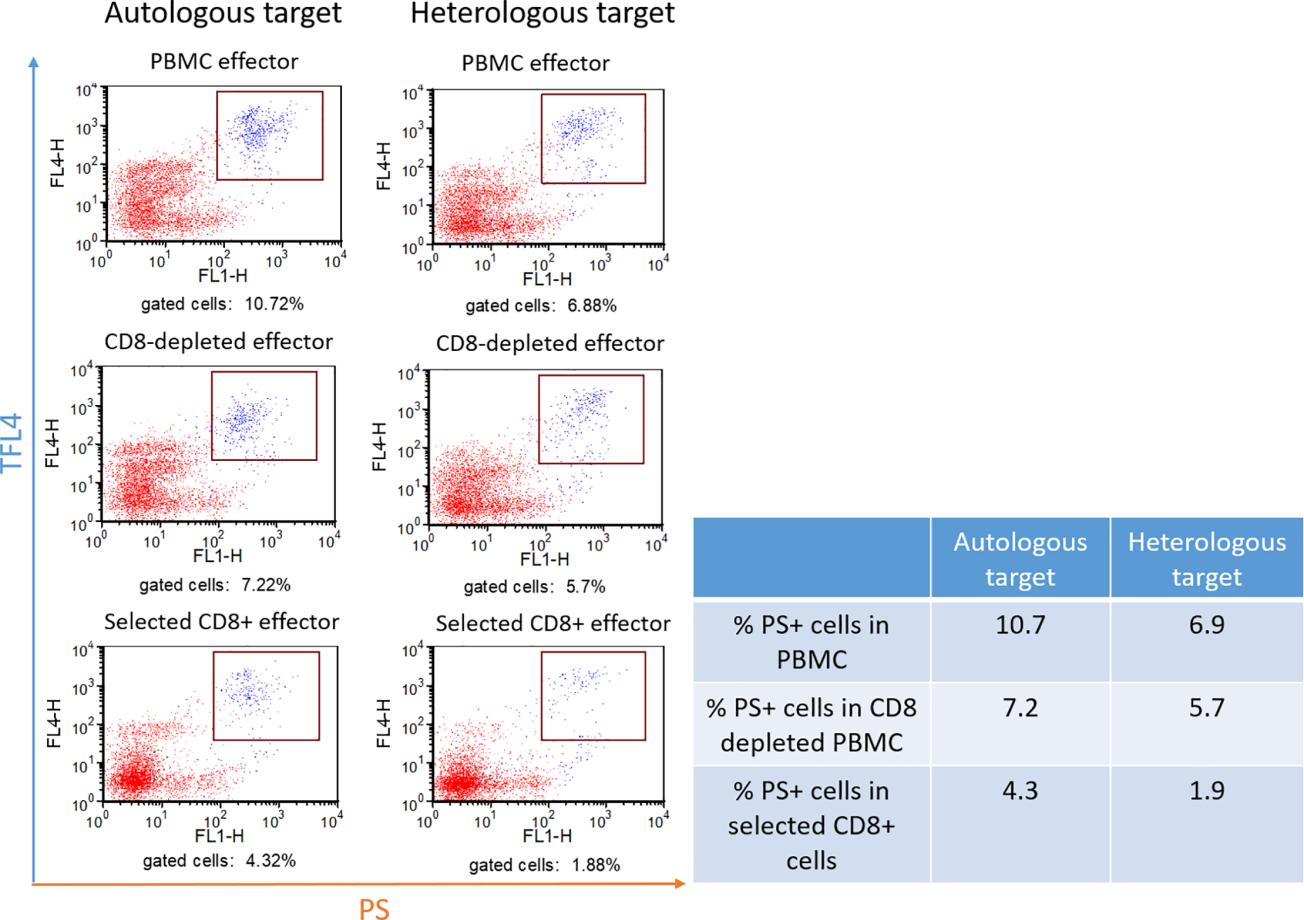
Effect of CD8^+^ T-cell depletion and selection on the killing of MDM infected with PRRSV for 3 hours which is before *de novo* synthesis of PRRSV proteins. The CTL assay was carried out using autologous (gilt-2) or MHC-matched heterologous (gilt-678) MDM infected with PRRSV_SD23983_ (MOI 19.3) and gilt-2 effector cells. Cytotoxic T-lymphocyte responses in PBMC, CD8^+^ T-cell-depleted or CD8^+^ T-cell-selected subpopulations of PBMC from gilt-2 were measured by the percentage MDM target cells (TFL4^+^) having death signals (PS^+^) at 3 hours post-PRRSV infection.

MDM target cells infected with PRRSV_23983_ for 3 hours and gilt-2 PBMC effectors depleted of CD8^+^ T-cells and selected CD8^+^ T-cells were used to determine the role of CD8^+^ T-cells in killing infected MDM early after infection. The depletion of CD8^+^ T-cells from PBMC reduced the killing of autologous MDM by 33% (Fig 6, panels 1 and 3; 10.72% with PBMC to 7.22% with PBMC depleted of CD8^+^ T-cells) and the killing of MHC-matched heterologous MDM by 17% (Fig 6, panels 2 and 4; 6.88% with PBMC to 5.70% with PBMC depleted of CD8^+^ T-cells). Selected CD8^+^ T-cells replaced only 40% of the killing of autologous target cells and 27% of the killing of heterologous MHC class I-matched target cells (Fig 6, panels 5 and 6: 4.32% and 1.88% with selected CD8+ T-cells using autologous and heterologous target MDM, respectively). These data demonstrated that some killing of MDM target cells at 3 hours after PRRSV infection was caused by CD8^+^ CTL from gilt-2, but that these CTL were not the major T-cell subpopulation involved in the killing at this time point.

### Identification of the predominant T-cell phenotypes with granzyme-B in the cytotoxic assay using MDM infected with PRRSV at different times

**Fig 7:**
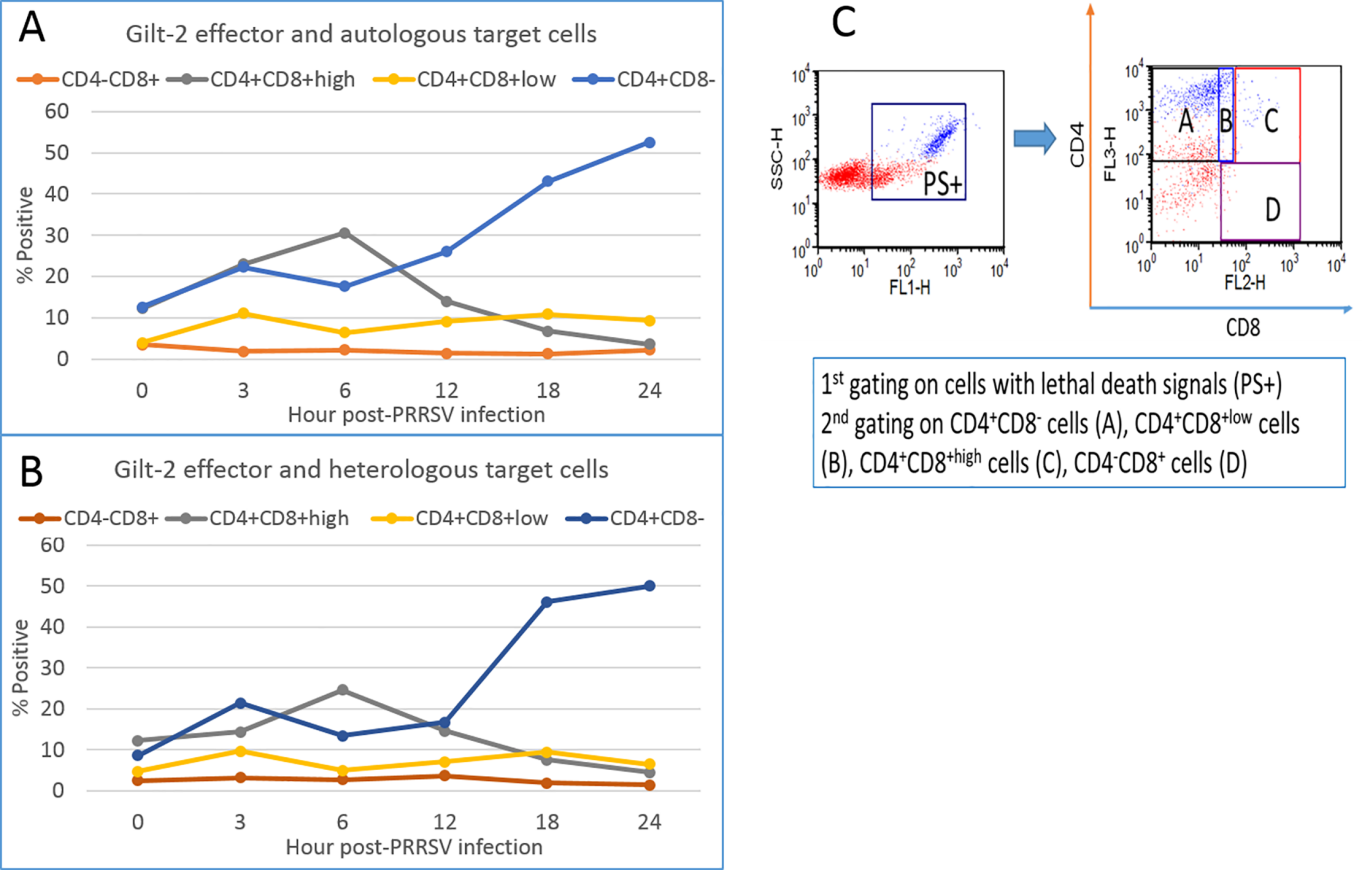
CD4^+^CD8^-^ and CD4^+^CD8^+high^ phenotypes have biphasic and monophasic cytotoxicity kinetics against PRRSV-infected MDM, respectively. The percentages of four T-cell phenotypes with PS^+^ death signals in the CTL assays using autologous (A) and heterologous (B) MDMs infected with PRRSV_SD23983_ for 0 to 24 hours and then incubated for 1-hour with gilt-2 effectors are presented in graphs. The gating strategies for different T-cells are presented (C). Autologous and heterologous MHC-matched MDMs were prepared from PBMC of gilt-2 and gilt-678, respectively.

Further studies were conducted to determine the percentage of four phenotypes (CD4^+^CD8^-^PS^+^, CD4^+^CD8^+low^PS^+^, CD4^+^CD8^+high^PS^+^, and CD4^-^CD8^+all^PS^+^) with killing activity at the different time points examined during the 24-hour time frame of the study. The gating strategy described in Fig 7C was used to obtain the data. The predominant gilt-2 CTL phenotypes activated during the biphasic presentation of epitopes on PRRSV-infected autologous MDM were CD4^+^CD8^+high^PS^+^ in early (3–6 hpi) and CD4^+^CD8^-^PS^+^ in both early (3 hpi) and late (18–24 hpi) time points (Fig 7A). Similar results were obtained with heterologous MHC-matched MDM targets (Fig 7B). The CD4^+^CD8^+low^PS^+^ T-cells responded weakly with an early and late peak of activity. No activity was observed with CD4^-^CD8^+^PS^+^ T-cells at any time point tested (Fig 7A & 7B).

## Discussion

The replication of PRRSV after entry into macrophages using CD163 as receptors [48–51] causes immune modulations in pigs, resulting in weak adaptive immune responses as well as persistence of the virus [38,52,53]. In spite of these challenges, PRRSV in blood, lung and lymphoid tissues of infected pigs is occasionally controlled before appearance of virus-specific neutralizing antibodies and this phenomenon strongly suggests a contribution of CMI in controlling the virus [31–34].

In this study, a virus suppression assay was developed to be used in determining if PBMC from gilts that recovered from infection with an intra-nasal dose of PRRSV could reduce the number of PRRSV-infected MDM. Total PBMC from gilts with two different MHC haplotypes and four subpopulations including CD4^+^- and CD8^+^-depleted PBMC and positively selected CD4^+^ and CD8^+^ T-cells were evaluated and shown to consistently reduce PRRSV-infected MDM by over 80%, with a high of 95.5%. Subsequent experiments were conducted with a flow cytometry assay that detects granzyme-B-mediated killing of target cells by CTL to determine if reduction in infected MDM was mediated by granzyme-B. The cytotoxic assay demonstrated that PBMC with suppressive activity also killed infected MDM and thus the major mechanism reducing PRRSV in the virus suppression assay was cytotoxicity. Both CD4^+^ and CD8^+^ CTL were shown to contain granzyme-B that was introduced into infected MDM. Analysis of killing over a 24-hour time course revealed killing was biphasic with two peaks detected at 3–6 hours and 16–24 hrs. These two peaks of killing were associated with two different T-cell phenotypes. Two CTL phenotypes including CD4^+^CD8^+high^PS^+^ and CD4^+^CD8^-^PS^+^ killed target cells infected for 3–6 hours which was possibly before de novo synthesis of virus [54]. However, the phenotype killing 24-hour infected target cells was only CD4^+^CD8^-^PS^+^. There were almost no CD4^-^CD8^+^PS^+^ CTL and very few CD4^+^CD8^+low^PS^+^ CTL at any time point after target cell infection. CD4^+^ CTL has not been reported in PRRSV-infected pigs although lack of CD8^+^ T-cell contribution in CMI responses against PRRSV in infected pigs was suggested in a previous study [55]. However, CD4^+^ CTL was reported in humans vaccinated with gp160 subunit protein of human immunodeficiency virus [56]. Further, Nef epitope-specific HLA-DRB1*0803-restricted CD4^+^ CTL in another study had strong gamma interferon production after stimulation with autologous B-lymphoblastoid cells infected with recombinant vaccinia virus expressing Nef or pulsed with heat-inactivated virus particles. Nef-specific CD4^+^ CTL exhibited strong cytotoxicity and viral suppression activity against both HIV-1-infected macrophages and CD4^+^ T-cells [57]. Helper functions of CD4^+^CD8^-^ T-cells for classical CD4^-^CD8^+^ CTL (T-helper 1) and B-lymphocytes (T-helper 2) have been clearly defined in many species including pigs [58, 59]. However, cytotoxic CD4^+^CD8^-^ T-cells to PRRSV has not been reported before this manuscript.

The killing of target cells by CD4^+^ and CD8^+^ CTL could have been due to two typical phenotypes (CD4^+^CD8^-^ or CD4^-^CD8^+^) or to CTL expressing both CD4^+^ and CD8^+^, as memory T- cells expressing both occur with a relatively high frequency in pigs [58–60]. Due to double positive memory T-cells increasing with age of pigs [58], the usual dichotomy between helper CD4^+^CD8^-^ T-lymphocytes and cytotoxic CD8^+^CD4^-^ T-lymphocytes may not occur. Possible contribution of natural killer (NK) cells and NKT cells in eliminating PRRSV-infected target cells observed in the virus suppression assay (VSA) cannot be completely disregarded. However, non-PRRSV-specific killing of target cells by NK and NKT cells should be minimal based on no significant killing in negative control PRRSV-free wells in VSA and CTL assay using the same effectors. This feature in pigs requires identifying the phenotype of T-cells causing cytotoxicity and other protective responses to systematically dissect protective mechanisms in PRRSV-infected and vaccinated pigs. Functional CTL responses were identified in pigs infected with classical swine fever virus (CSFV) and foot and mouth disease virus [61,62]. Major phenotype of functional CTL in CSFV-infected miniature pigs were CD3^+^CD4^-^CD8^+high^ T-cells [61,63]. However, CD3^+^CD8^+high^ T-cells in peripheral blood of PRRSV-infected pigs were functionally impaired in CTL assay suggesting lack of typical CTL phenotype [31]. In addition, limited information on MHC class-I molecules presenting specific PRRSV epitopes hinder developing diverse MHC class-I-epitope-specific tetramers for rapid quantitation of PRRSV-specific T-cell responses. Even if pig MHC class-I-epitope-specific tetramers become available for PRRSV, tetramer-based quantitation is limited by the lack of information it provides about the function of positive cells and their protective efficacy. Therefore, developing functionally relevant CMI assays using natural target cells for PRRSV replication and the measurement of terminal effector function (e.g., virus suppression and/or cytolysis) as used in this study will be crucial for defining the protection correlates in PRRSV-infected and or vaccinated pigs.

Another important finding in this study is that on the day of PBMC collection, the three gilts with highly suppressive effector T-cells had weak and no detectable neutralizing antibody responses against challenge PRRSV_SD23983_ (1:4–1:16 virus neutralization titer) and a phylogenetically close PRRSV_VR-2332_ (<1:4), respectively [64]. This observation additionally suggests that PRRSV-specific CTL might be the major contributor for PRRSV control with no assistance of neutralizing antibodies. To extend these findings, the PRRSV epitopes recognized by the two different CTL phenotypes need to be defined and it needs to be determined whether these CTL are MHC class I- or class II restricted. For these new findings to impact vaccine strategies, it will be important to determine if the predominant CD4^+^CD8^-^ CTL recognizing PRRSV epitopes presented both early and late are more effective in the control of PRRSV than the CD4^+^CD8^+high^ CTL limited to the early stage of PRRSV replication or if both are needed for virus control.

## Conclusions

PRRSV-specific CTLs were found in blood from pigs that had recovered from PRRSV infection that could kill PRRSV-infected cells *in-vitro*. A phenotype of CTL (CD4^+^CD8^+high^) killed infected cells within 3–6 hours after cell infection and another phenotype (CD4^+^CD8^-^) killed infected cells at 16–24 hours after infection. The finding of these CTL in pigs, that had recovered from PRRSV infection, suggests that vaccines can be developed that elicit both CTL and neutralizing antibody that would be more effective than current vaccines.

## Materials and methods

### Ethics statement

All experiments involving animals were carried out in accordance with the recommendations in the Guide for the Care and Use of Laboratory Animals of the National Institutes of Health and in conformance with the United States Department of Agriculture animal research guidelines, under a protocol approved by the Washington State University Institutional Animal Care and Use Committee (ASAF # 04372–002).

### Propagation and titration of PRRSV

The SD23983 strain of PRRSV (60) was propagated in the agent-free MARC-145 cell line (American Type Cell Collection, Manassas, VA, USA), cultured in complete Eagle’s Modified Essential Medium (EMEM; Corning, Manassas, VA, USA) supplemented with 10% fetal bovine serum (FBS; Seradigm, Radnor, PA, USA) at 37°C. Confluent monolayers in 150 cm^2^ flasks were inoculated with a multiplicity of infection (MOI) of 0.01. When the virus-induced cytopathic effect (CPE) reached the maximum at 5 to 7 days, culture supernatants were collected and centrifuged at 4,000 × *g* for 10 minutes at 4°C to eliminate cell debris.

The titer of PRRSV in each supernatant pool was determined using the microtitration infectivity assay [65]. The median tissue culture infective dose (TCID_50_) was calculated according to the method of Reed and Munch http://ntserver1.wsulibs.wsu.edu:2091/science/article/pii/S037811351200034X-bib0130[66]. Briefly, confluent monolayers of MARC-145 cells, prepared in 96-well plates (Corning), were inoculated with 10-fold serial dilutions of PRRSV prepared in EMEM. The count of CPE present after seven days of culture was used to calculate the TCID_50_/mL.

### PRRSV-infected and uninfected gilts used in this study

Five 12-month-old gilts (Sus scrofa domesticus) born from the same dam were selected and tested by PCR and or indirect immunofluorescent antibody to verify they were free of PRRSV, porcine circovirus type-2, porcine epidemic diarrhea virus and other major porcine pathogens. Three of the five gilts (gilt-1, gilt-2, gilt-3) were intra-nasally infected with 1.58 x 10^4^ TCID_50_ of PRRSV_SD23983_ and were maintained in isolation rooms. The infected gilts were the source of PBMC for the virus suppression and CTL assays reported in this study. The other two gilts (gilt-676 and gilt-678) were kept in a separate room to serve as a source of uninfected MHC-matched heterologous monocyte derived macrophage target cells.

### MHC class I and II typing of gilts

Each of five gilts used in this study were genotyped for three swine leukocyte antigen (SLA) class I (SLA-1, -2, -3) and three SLA class II (DRB1, DQB1, DQA) genes using the low-resolution (Lr) polymerase chain reaction with sequence-specific primer (PCR-SSP) typing panels as previously described [67,68]. SLA class I and class II haplotypes were deduced based on the published (15713212, 16305679, 18760302, 19317739) and unpublished haplotypes identified in outbred commercial pigs (http://www.jimmunol.org/cgi/content/meeting_abstract/186/1_MeetingAbstracts/170.1). Gilts 676, 1 and 3 had a fully-matched set of SLA class I and II haplotypes (Table 1). Gilts 678 and 2 had another fully-matched set of SLA class I and II haplotypes (Table 1).

**Table 1:** Major histocompatibility complex (MHC) genotypes of gilts used in this study.

	PRRSV challenge	MHC class I haplotype	MHC class II haplotype	MHC matching
Gilt-1	SD23983, intranasal	Lr-32.0/35.0	Lr-0.1/0.12	MHC-matched with gilts 3 and 676
Gilt-2	SD23983, intranasal	Lr-4.0/39.0	Lr-0.2/0.23	MHC-matched with gilt-678
Gilt-3	SD23983, intranasal	Lr-32.0/35.0	Lr-0.1/0.12	MHC-matched with gilts 1 and 676
Gilt-676	None	Lr-32.0/35.0	Lr-0.1/0.12	MHC-matched with gilts 1 and 3
Gilt-678	None	Lr-4.0/39.0	Lr-0.2/0.23	MHC-matched with gilt-2

Genotypes in this table were determined for three swine leukocyte antigen class I (SLA-1, -2, -3) and three class II (DRB1, DQB1, DQA) genes using the low-resolution (Lr) polymerase chain reaction with sequence-specific primer typing panels.

### Preparation and storage of PBMC

Swine PBMC were isolated from fresh venous blood collected in 5 mM EDTA (final concentration) by centrifugation on a discontinuous gradient using Lymphoprep™ (STEMCELL Technologies, Vancouver, Canada) [52]. Briefly, blood in 5 mM EDTA was diluted two-fold in 10mM phosphate-buffered saline (PBS, pH 7.4) containing 20% anti-coagulant citrate dextrose (ACD) and 2% FBS. Thirty-five milliliters of the diluted blood was added to a SepMate™-50 tube (STEMCELL Technologies) containing 15 mL of Lymphoprep™. The tubes were centrifuged at 1,100 × *g* for 10 minutes. The buffy coat cells, at the supernatant cell interface, were collected and pelleted by centrifugation. The cells were resuspended in PBS containing 20% ACD and 2% FBS, and subjected to cycles of centrifugation until platelets were removed. Isolated PBMCs were counted, resuspended in a freezing medium containing 10% dimethyl sulfoxide (J.T. Baker, Center Valley, PA, USA) and 90% FBS, and stored in liquid nitrogen until used.

### Preparation of monocyte-derived macrophages (MDM)

Frozen PBMC were thawed, washed with serum-free EMEM (Corning, Manassas, VA, USA), and incubated in serum-free EMEM at 37 ^o^C for 2 hours in 100 cm petri dishes. The culture medium and non-adherent cells were discarded. The adherent cells were cultured in RPMI-1640 medium (Life Technologies, Carlsbad, CA, USA) supplemented with 10% FBS and 5 ng/ml of murine macrophage-colony stimulating factor (Biolegend, San Diego, CA, USA) every other day for 6 days. Six-day-old MDM were used as the target cells for virus suppression and CTL assays. MDM pulsed with heat inactivated PRRSV were used to stimulate a recall response in PBMC obtained from gilts that had recovered from infection.

### Preparation of PRRSV-specific T-cell-enriched PBMC

PRRSV-specific T-cell-enriched cultures were generated by modification of a previously described method [52]. Briefly, six-day cultures of MDM prepared in 150 cm^2^ tissue culture flasks were pulsed with heat-inactivated PRRSV for two hours. PBMC were resuspended to make 10^6^ cells per ml in 40 ml, then added to the flask with heat-inactivated PRRSV-pulsed MDM and cultured for 7 days in RPMI-1640 medium supplemented with 10% of FBS and 5 ng/ml of recombinant porcine IL-7 (R&D Systems, Minneapolis, MN, USA).

### Positive and negative selection of T-cell subpopulations in PBMC

PBMC alone, PBMC depleted of CD4^+^ or CD8^+^ T-cells, and positively selected CD4^+^ and CD8^+^ T-cells were prepared to conduct the suppression and CTL assays. MAbs (Washington State university Monoclonal Antibody Center, WSUMAC http://vmp.vetmed.wsu.edu/resources/monoclonal-antibody-center) specific for CD4^+^ (PT90 IgG2a) and CD8^+^ (PT81B IgG2b) T-cells were used with magnetic beads (Miltenyi, Cologne, Germany) to obtain the different populations of cells according to the manufacturer’s instructions. Briefly, PBMC were added to 50 μL of mAbs specific for CD4^+^ or CD8^+^ (10 μg/reaction) per 10^6^ million, and then incubated at 4 ^o^C for 30 minutes with gentle agitation every ten minutes. The cells were washed twice with PBS and then mixed with magnetic beads coated with goat anti-mouse IgG2a/IgG2b (20 μL of magnetic bead suspension per 10^7^ cells) and incubated at 4 ^o^C for 15 minutes. The cells were passed through a column placed on a magnetic stand. The pass-through cells were collected as the negatively selected population and the cells on the column, collected as the positively selected population. Flow cytometric examination of aliquots of the different populations, following labelling with the respective mAbs, showed the purity of the negatively and positively selected populations were >95%.

### Virus suppression assay

The virus suppression assay (VSA) measured the reduction of PRRSV replication in MDM by PRRSV-specific T-cells and their subpopulations. MDM target cells were generated from PBMC of gilts that had recovered from clinical infection (source of autologous target cells), as well as from PBMC of PRRSV-free gilts (source of MHC-matched heterologous targets). In a preliminary validation experiment, 6-day cultured MDMs were highly susceptible for PRRSV infection (supporting data Fig 1) and stable that are critical features for target cells in VSA and cytotoxicity assay. Six-day cultures of MDM were harvested using a cell dissociation solution (Sigma-Aldrich, St. Louis, MO, USA). The harvested MDMs were infected with PRRSV (MOI 0.00005) and incubated with antigen-stimulated PBMC, PBMC depleted of CD4^+^ or CD8^+^ T-cells, and positively selected preparations of CD4^+^ or CD8^+^ T-cells. PBMC effector to target ratio was 5:1 (5 x 10^6^ effector cells and 10^6^ MDM). The positively selected CD4^+^ and CD8^+^ T-cell effector to target ratio was 0.5:1 (0.5 x 10^6^ effector cells and 10^6^ target MDM). Target and effector cells in VSA wells of 24-well plates were maintained in complete MEM (cMEM) containing 0.5 ng/ml of recombinant porcine IL-2 (R&D Systems, Minneapolis, MN, USA) for 6-days. cMEM was refreshed every other day. To determine the suppression of PRRSV replication in MDM by effector cells, infected MDM were labelled with mAbs specific for CD172a (74-22-15 clone, IgG1 isotype, WSUMAC) and PRRSV-N (2D6 clone, IgG2b isotype; VMRD) and enumerated by flow cytometry 6-day post infection. The additional mAbs and secondary antibody conjugates used to phenotype the effector populations included anti-CD8 (PT81B clone, IgG2b, WSUMAC), anti-CD4-PerCP-Cy5.5 (BD Pharmingen, San Jose, CA, USA), goat anti-mouse IgG1-FITC (Life Technologies) and goat anti-mouse IgG2b-R-PE (Life Technologies). The labelled cells were analyzed with a FACS Calibur analytical flow cytometer (Becton Dickinson Immunocytometry systems, San Jose, CA, USA). Briefly, a gate was placed on MDM based on forward and side light scatter (FSC vs SSC), and then the suppression of PRRSV replication was calculated by percent reduction of the PRRSV-infected MDM (CD172a^+^PRRSV-N^+^ cells and CD172a^**-**^PRRSV-N^**+**^ cells) in the presence of effectors as compared to target cells alone. The reason for inclusion of CD172a-negative PRRSV-infected MDM in the calculation of % suppression is related to the phenomenon that many surface molecules including CD172a, MHC class I and II are down-regulated on porcine macrophages at 12 or longer hour post-infection [69] as observed in other virus-infected targets such as simian immunodeficiency virus-infected CD4 T-cells [44, 45]. The percent reduction of infected MDM was calculated as evidence for the suppression of PRRSV replication by effector T-cells. Highly effective virus suppression was defined as 80% or more reduction of PRRSV-infected MDM in the presence of effectors as compared to target cells alone. The rationale for using a very low MOI (0.00005) of PRRSV in this VSA is to detect CTL responses sensitively in a physiologically more relevant condition to natural PRRSV infection to pigs.

### CTL assay

A flow cytometric CTL assay was designed to measure the killing of PRRSV-infected MDM by CTLs. Target MDM cells were generated from PBMC of gilts that had recovered from PRRSV-infection as described above (autologous target cells), as well as PBMC from PRRSV-free gilts (MHC-matched heterologous target cells). Six-day cultures of MDM in cMEM were infected with 19.3 MOI of PRRSV and cultured for 0, 3, 6, 12, 18 and 24 hours. Any remaining PRRSV in the culture medium after incubation were removed by washing with PBS (pH 7.4). PRRSV-infected MDM were labelled with TFL4, a fluorescent permeable viable dye to distinguish live target cells from effector cells, per manufacturer’s protocol (OncoImmunin Inc., Gaithersburg, MD, USA). The TFL4-labelled MDM were washed to remove excess dye and then incubated with total PBMC or T-cell subpopulations for 0, 3, 6, 12, 18 and 24 hours. At the designated time points MDM were brought into suspension and then pelleted by centrifugation at 800 x *g* for 10 minutes. The effector to target ratio was 5:1 (0.5 x 10^6^ effectors and 0.1 x 10^6^ MDM). Target and effector cells were incubated in wells of a 96-well plate with a cell permeable fluorogenic substrate for granzyme-B (GzB) and upstream caspases (PanToxiLux substrate (PS), OncoImmunin Inc.) for 1 hour at 37 ^o^C. Following incubation, the cells were washed twice with PBS. Surface staining of effector CD4^+^ T-cells was carried out using CD4-PerCP-Cy5.5 (BD Pharmingen). For CD8^+^ T-cells, cells were reacted with anti-CD8 PT81B (WSUMAC) followed by staining with goat anti-mouse IgG2b antibody conjugated to R-PE (Life Technology). To analyze the killing of infected MDM mediated by effector cells, dead cells were detected by fluorescence of the fluorogenic substrate by flow cytometry. Briefly, a selective gate was placed on MDM in FSC vs SSC to exclude background. Cell populations were then visualized in fluorescent channels to distinguish labelled target cells from non-fluorescent effector cells. The percentage of target cells killed by effector cells was determined by enumerating TFL4^+^PS^+^ MDM [47,70]. Activated CTL phenotypes were identified as CD4^+^CD8^-^PS^+^, CD4^+^CD8^+low^PS^+^, CD4^+^CD8^+high^PS^+^, and CD4^-^CD8^+all^PS^+^.

## Supporting information

S1 Fig Six-day cultured monocyte-derived macrophages are highly susceptible for PRRSV infection using magnetic nanoparticles. (DOCX)
