# Supplementary material for: Pigs that recover from porcine reproduction and respiratory syndrome virus infection develop cytotoxic CD4+CD8+ and CD4+CD8- T-cells that kill virus infected cells
Source: PLoS One. 2018 Sep 6;13(9):e0203482. doi: 10.1371/journal.pone.0203482 (PMC6126854; doi:10.1371/journal.pone.0203482)

Supporting Information: S1 Fig. Six-day cultured monocyte-derived macrophages are highly susceptible for PRRSV high MOI infection using magnetic nanoparticles.


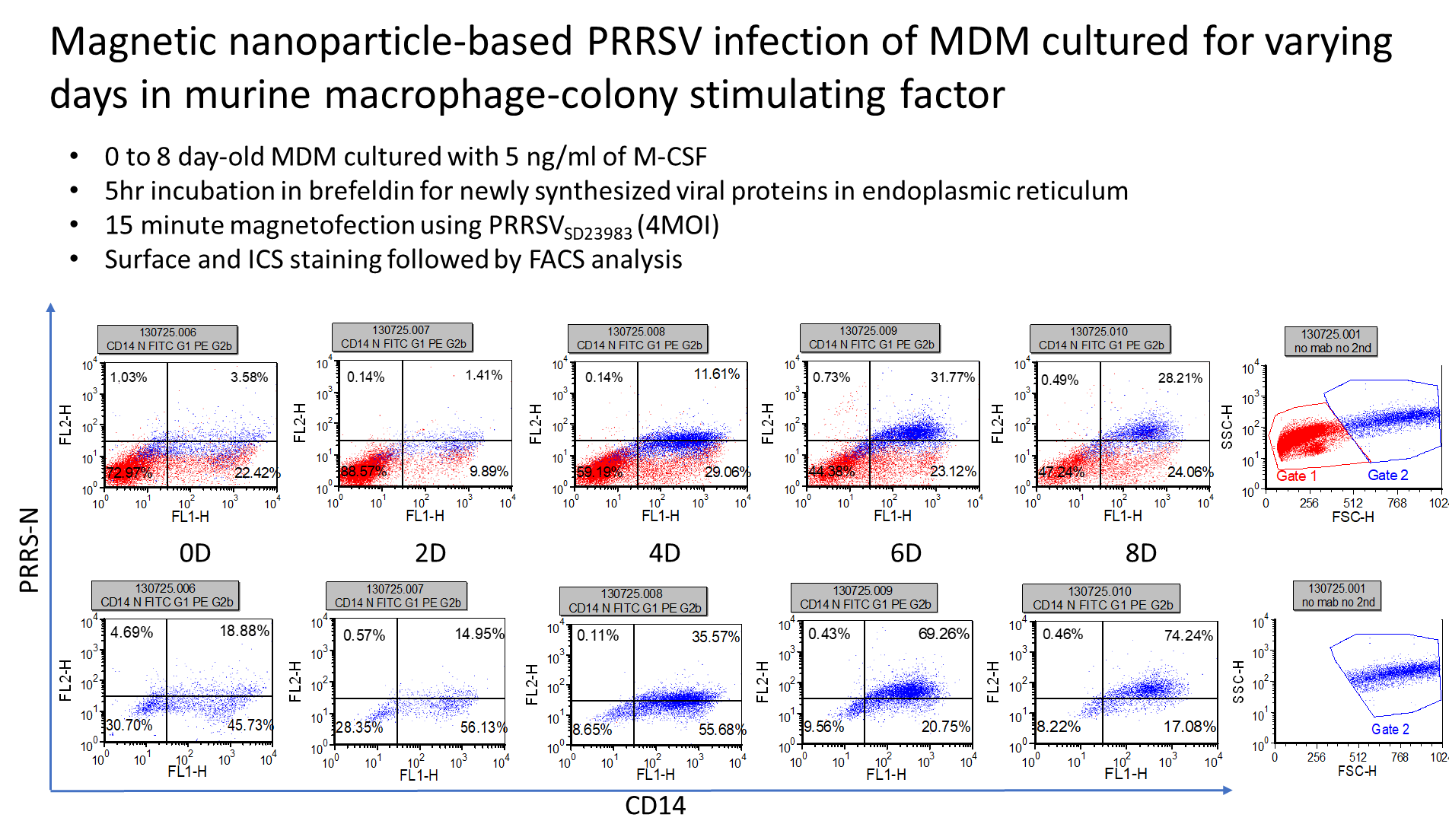

Supplement: S1 Fig — (DOCX) [file pone.0203482.s001.docx]
